# Contested science: Individuals with higher metacognitive insight into interpretation of evidence are less likely to polarize

**DOI:** 10.3758/s13423-021-01993-y

**Published:** 2021-10-29

**Authors:** Nadia Said, Helen Fischer, Gerrit Anders

**Affiliations:** 1 Department of Psychology, University of Tübingen, Tübingen, Germany; 2 Interdisciplinary Center for Scientific Computing (IWR), Heidelberg University, Heidelberg, Germany; 3 Max Planck Institute for Human Development, Center for Adaptive Rationality, Berlin, Germany

**Keywords:** Belief updating, Metacognition, Polarization, Politicized science, Motivated reasoning

## Abstract

Societal polarization over contested science has increased in recent years. To explain this development, political, sociological, and psychological research has identified societal macro-phenomena as well as cognitive micro-level factors that explain how citizens reason about the science. Here we take a radically different perspective, and highlight the effects of metacognition: How citizens reason about their own reasoning. Leveraging methods from Signal Detection Theory, we investigated the importance of metacognitive insight for polarization for the heavily contested topic of climate change, and the less heavily contested topic of nanotechnology. We found that, for climate change (but not for nanotechnology), higher insight into the accuracy of own interpretations of the available scientific evidence related to a lower likelihood of polarization over the science. This finding held irrespective of the direction of the scientific evidence (endorsing or rejecting anthropogenicity of climate change). Furthermore, the polarizing effect of scientific evidence could be traced back to higher metacognitive insight fostering belief-updating in the direction of the evidence at the expense of own, prior beliefs. By demonstrating how metacognition links to polarization, the present research adds to our understanding of the drivers of societal polarization over science.

**Supplementary Information:**

The online version contains supplementary material available at 10.3758/s13423-021-01993-y.

## Introduction

Belief polarization, the tendency of individuals to update prior beliefs in opposing directions after observing the same evidence (Cook & Lewandowsky, 2016), has long been observed in the context of issues of worldview such as immigration, multiculturalism or religion (Dixit & Weibull, 2007). Recently, however, citizens have become polarized over seemingly emotionless science. Research into the “widening gap” has demonstrated that, despite increasing scientific consensus, partisan polarization over climate change has increased among American voters since the late 1990s, and continues to do so (Dunlap et al., 2016; Dunlap & McCright, 2008). Similar polarization trends have been observed among the American public for stem cell research and human evolution (Drummond & Fischhoff, 2017), or vaccination (Schmidt et al., 2018). A multitude of research has identified societal macro phenomena and “mega trends” to reinforce societal belief polarization, such as the rise of the Internet and social media (Gabler, 2016; Sunstein, 2018)), as well as micro-level cognitive factors such as prior beliefs about the science (Fryer Jr et al., 2019; Jern et al., 2014; Kahan et al., 2017), and biases in information processing, such as confirmation bias (Del Vicario et al., 2017), or motivated reasoning (Hart & Nisbet, 2011).

What these explanations, as diverse as they may be, have in common, is that they try to isolate drivers of how citizens reason *about the science*. Previous explanations therefore focused on object-level cognition, reasoning about the *subject matter* itself. Here we take a radically different perspective, highlighting the importance of metacognition*:* How citizens reason about *their own reasoning.* That is, the present research illuminates the role of insight into the validity of our own thoughts. For example, on the object-level, citizens might reason that a novel piece of evidence about climate change is inaccurate because it contradicts own prior beliefs. On the metacognitive level, in contrast, citizens can take a step back, and reflect on how much their own reasoning about the evidence is distorted by prior beliefs, and hence lacking validity. Importantly, we explore whether such critical examination of own interpretations can directly affect group polarization: Are individuals with higher metacognitive insight into the validity of their own interpretations of the available evidence less likely to polarize over the science?

Metacognitive reflection on our thoughts can lead to enhanced humility about their validity: While object-level reflections take our interpretations of the world for granted, metacognitive reflections enable us to distance ourselves from our interpretations and recognize them as what they are – interpretations – and, consequently, as potentially incorrect (Frith & Frith, 2012). Such insight about the fundamental fallibility of our interpretations (that, ultimately, our beliefs are built upon) may be a necessary precondition to be appropriately attentive to, and open for, belief-disconfirming evidence. Lacking insight into the validity of one’s thought, in contrast, manifests itself in unjustified confidence in interpretations of the world, and can propel the psychological tendency to interpret evidence selectively such that it conforms to prior beliefs (Meyer et al., 2013). Unjustified confidence in own positions and judgments also tends to be enhanced in individuals holding radical political beliefs (Rollwage et al., 2018; Zmigrod et al., 2019), and lacking updating of confidence in one’s beliefs after receiving corrective feedback is related to right-wing authoritarianism (Sinclair et al., 2020). Recent neuroscience research has identified modulated neural processing as a key mechanism behind confidence-driven effects in that higher confidence amplifies selective integration of confirmatory evidence, while confirmatory evidence is abolished (Rollwage et al., 2020). When citizens are confronted with information on politicized science, lacking insight into the validity of their interpretation of the evidence might therefore foster the selective, belief-congruent interpretation of this evidence.

Critically, these metacognitive effects could then drive polarization over contested science: If citizens lacking metacognitive insight into their reasoning about the available evidence tend to update their beliefs in the direction of their prior beliefs, they should be a stronger driver of group polarization over the science. Conversely, if citizens with more accurate insight into their interpretation update their beliefs less in the direction of their prior beliefs, this should reduce group polarization. While previous research has investigated whether citizens have insight into their belief change (Wolfe & Williams, 2018), that is, the *outcome*, we investigated whether citizens have insight into their evidence interpretation, that is, the *process*. This is a crucial distinction since being aware of the fallibility of own reasoning processes may enhance potential corrections of this process while still *forming beliefs,* as opposed to already having formed them.

To sum up, we propose that metacognitive insight about the validity of our interpretations of evidence is recognizing that these interpretations may be incorrect, and can reduce the likelihood of group polarization over the topic in question.

## Method

The data supporting all analyses, figures, and tables as well as the analysis code (R) can be found at: 10.6084/m9.figshare.13139351

### Participants

A total of N = 354 participants completed the experiment. Participants were recruited via Mturk, an online participant pool that provides samples that are reasonably representative of the general US population (McCredie & Morey, 2019). A total of n = 189 (53%) participants were female, and the average age of participants was 42 years (range = 21–75). Participants had a range of educational backgrounds (lowest school-leaving certificate: 1%; middle school-leaving certificate: 12%; highest school-leaving certificate: 28%; Bachelor’s degree: 43%; Master’s degree or equivalent: 14%; other: 0.8%).

### Materials

Participants read several texts summarizing scientific evidence about the topics climate change and nanotechnology. The summaries varied the *direction of evidence* as either endorsing or rejecting (a) the anthropogenicity of climate change (two texts endorsing, two rejecting^1^), and (b) the benefits of nanotechnology, relative to its risks (four on benefits, four on risks). The absolute number of endorsement/rejection texts was equal within each topic, such that the provided evidence base was balanced. The topics of nanotechnology and climate change both constitute science topics that are contested, but to a different degree since nanotechnology constitutes a less contested topic compared to climate change (Cobb & Macoubrie, 2004; Bertoldo et al., 2016; Drummond & Fischhoff, 2017). Hence, comparing climate change and nanotechnology in one study allows us to explore whether metacognitive insight as a driver of polarization depends on the degree of polarization of the science.

The summaries on climate change were taken from previous research (Fryer Jr et al., 2019). The summaries for nanotechnology were newly developed for the present study, and pre-tested by a sample of N = 49 psychology students at Heidelberg University to ensure the displayed direction of evidence (endorsement vs. rejection) could be classified as intended. All summaries were of approximately equal length (around 100 words each), comprehensible without expert knowledge, and consisted of three parts, providing: (1) a description of the study or general introductory sentence; (2) a summary of the results; and (3) the conclusions that can be drawn from the study. All summaries are provided in the Online Supplementary Material.

### Procedure

We employed four different control mechanisms to ensure reliable and valid assessment. First, we only accepted Mturk workers with a 98% or higher approval rating. Second, before entering the survey, participants completed a captcha to exclude potential bots. Third and fourth, we introduced three practice rounds that participants needed to complete before entering the main study. The practice rounds were completed on an unrelated topic (gluten sensitivity), and comprised (a) attention checks as well as (b) task-comprehension checks.

The main study was completed in the following order for both topics (climate change and nanotechnology): (i) prior beliefs; (ii) provision of each text summarizing scientific evidence, text-specific assessment of the (iia) interpretation of each text, and (iib) confidence in the accuracy of the interpretation; (iii) posterior beliefs; (iv) demographics (age, education, and gender).

Order of the topics, as well as the summaries within each topic, were randomized. Each summaries was displayed on a separate page, and the respective text-specific questions (interpretation of the text, confidence in interpretation) were displayed on the consecutive page, so that participants needed to answer from memory. This was done to ensure that the questions assessed actual interpretation, including potential distortions of the evidence, rather than a simple look-up of the information provided. Participants went through the survey at a self-paced speed.

### Measures

#### Prior and posterior beliefs

The same items were used to assess prior and posterior beliefs. For climate change, participants indicated: “Do you think human activity is the cause of increasing temperatures?” on a 17-point scale ranging from from -8 (“I think that human activity is NOT the cause of increasing temperatures”) to 8 (“I think that human activity is the cause of increasing temperatures”). For nanotechnology, participants indicated: “Do you think that the positive outcomes of nanotechnology outweigh the risks?” on a 17-point scale ranging from -8 (“I think that the risks of nanotechnology strongly outweigh the positive outcomes”) to 8 (“I think that the positive outcomes of nanotechnology strongly outweigh the risks”), for the texts on nanotechnology.

#### Interpretation of the direction of evidence

Participants indicated their interpretation of the direction of evidence for climate change, and nanotechnology, each on a 17-point scale. Specifically, participants indicated their interpretation “This summary provides evidence that...” on a scale from -8 (“human activity is NOT the cause of increasing temperatures”) to 8 (“human activity is the cause of increasing temperatures”) for climate change, and from -8 (“the risks of nanotechnology strongly outweigh the positive outcomes”) to 8 (“the positive outcomes of nanotechnology strongly outweigh the risks”).

#### Metacognitive confidence in interpretation

Participants provided item-specific confidence judgment by indicating, after each interpretation of each text: “How certain are you that your assessment is correct?” on a 6-point scale ranging from 50% (“I guessed”) to 100% (“I am certain”).

### Analysis

#### Task sensitivity

To measure the accuracy of the interpretation of the scientific evidence, we determined *task sensitivity d’* as specified in a Signal Detection Theory (SDT) framework by calculating the difference of the Z(True Positive) and the Z(False Positive) rate, with Z being the inverse cumulative density function of the normal distribution. Task sensitivity d’ reflects participants’ ability to correctly distinguish between evidence as endorsing or rejecting anthropogenicity and risks, respectively. Ratings above 0 were classified as endorsement, ratings below zero were rated as rejection. Since accuracy was coded only with respect to rejection versus endorsement, ratings of 0 were rated as incorrect

#### Metacognitive sensitivity

To measure metacognitive sensitivity, we determined *meta-d’* for each participant (Maniscalco & Lau, 2014). *Meta-d’* is a bias-free measurement of sensitivity in that it controls for metacognitive bias, participants’ general tendency to report high/low values of confidence. *Meta-d’* expresses metacognitive sensitivity in an SDT framework, and assesses the degree to which participants’ confidence judgments reflect accurate versus inaccurate object-level responses, controlling for their response bias. That is, in our case, metacognitive sensitivity reflects the degree to which participants differentiate between correct versus incorrect interpretation of the object-level evidence in their metacognitive judgment. To do so, *meta-d’* is defined as the object-level d’ that would be *expected* to have produced the observed confidence data given the same response bias *c*, and given ideal metacognitive sensitivity. *Meta-d’* can be interpreted as the sensory evidence available for the metacognitive task in the same signal-to-noise ratio units that measures *d’* as the sensory evidence available for the object-level task. Hence, *meta-d’* values of zero indicate metacognitive performance at chance level, values < 0 indicate metacognitive performance below chance levels, and values > 0 indicate metacognitive performance above chance level.

To illustrate, two participants could have the same task performance (d’ = 2) but two different values of metacognitive sensitivity (*meta-d’*_A_ = 1.9, *meta-d’*_B_ = 1.2). These participants would only differ in their metacognitive abilities: For participant A, confidence ratings are more often lower for incorrect answers and higher for correct answers, resulting in a higher meta-d’ (*meta-d’*_A_ = 1.9) compared to participant B, where confidence ratings reflect correct and incorrect answers to a considerably lower degree (*meta-d’*_B_ = 1.2).

To compute meta-d’, we used a hierarchical Bayes procedure (Fleming, 2017), and code provided at https://github.com/smfleming/HMeta-d. This analysis has the advantage of producing reliable measurements when trials numbers are low (at least in the case of group estimates; Fleming, 2017).

#### Influence of prior beliefs on interpretation of the evidence

To measure the influence of prior beliefs on the interpretation of the scientific evidence, linear regression models were employed following the analysis of Fryer Jr et al. (2019). Interpretation ratings were averaged, separately per direction of evidence, and domain. The regression analysis was performed separately for each direction of evidence. The equations are in the form:

1 $$ {Interpretation}_i=\alpha +\gamma\ {PriorBelief}_i+{\varepsilon}_{i.} $$

with the index *i* denoting individuals, *α* and *γ* denoting are the regression parameters, and *ε*_*i*._ denoting the uncertainty for individual *i*.

This analysis provides the unique benefit of delivering regression parameters that can be readily interpreted by providing direct psychological meaning. First, the constant α corresponds to the *average interpretation* of the summary, such that the direction of the value of the parameter (positive or negative) indicates the interpreted direction of the evidence (endorsement or rejection). That is, positive values of α signify that a text was interpreted as endorsement on average, negative values of α signify that a text was interpreted as rejection.

More importantly, second, the regression parameter γ corresponds to the direction and strength *of the influence of the prior belief on the interpretation of evidence*. Positive values signify the extent to which an interpretation is influenced in the direction of the prior belief, that is, the extent to which the prior belief enhances the interpretation of the evidence in the direction of the prior. Negative values signify the extent to which an interpretation is influenced in the direction opposite to the prior belief. Values of zero signify that the prior belief had no influence on the interpretation of evidence. For example, consider a participant who has a strong prior belief that nanotechnology benefits outweigh its risks. In this case, positive values of γ signify that evidencet supporting this belief has a reinforcing influence on that belief – that is, further strengthens the belief in the direction of the prior.

The regression analysis was performed in R employing the linear model-function (lm) (R Core Team, 2018). Errors are calculated using the ordinary least square (OLS)-method (Seabold & Perktold, 2010). To assess statistical significance of the difference of any two parameters (e.g., comparing two groups of participants), we compared their respective 3-σ regions. Disjoint σ regions reflect the probability that those parameters are not equal.

#### Proportion of polarizers

To estimate the proportion of polarizers, we identified individuals who became more extreme in their prior compared to their posterior beliefs. Participants who indicated a prior belief of zero were not included into the estimation of polarizers (see Fig. 2, sub-groups “opinion creation” and “polarizers” for the proportion of both sub-groups regarding the two topics). We then calculated the *proportion of polarizers G*_*pol*_ relative to all participants. The error was estimated as the relative Gaussian standard error.

*Do differences in task sensitivity and metacognitive sensitivity moderate the relationship of prior beliefs and evidence interpretation?* To analyze whether differences regarding task sensitivity (d’) and metacognitive sensitivity (meta-d’) moderate the relationship between prior beliefs and the interpretation of the evidence, participants were split into high and low scorers for each variable. Splitting participants into categories provides the unique advantage that the slope γ remains a psychologically interpretable variable, namely the influence of prior beliefs on the interpretation of the evidence. As a consequence, high and low scorers of task sensitivity and metacognitive sensitivity can be directly compared on the same scale to assess whether they differ in the extent to which the prior influences the interpretation of the evidence.

Individuals were categorized based on their individual value relative to the mean for each variable such that individuals with values higher than the mean were categorized as high scorer, and individuals with values lower than the mean as low scorer.

The influence of the variables P on the relationship of prior beliefs and evidence interpretation is calculated as follows:

2 $$ {Interpretation}_{i\in \mathrm{P}}={\alpha}_p+{\gamma}_p\ {PriorBelief}_{i\in \mathrm{P}}+{\varepsilon}_{ii\in \mathrm{P},} $$

where P denotes the subgroups for d’, and meta‐d’.

### Results

Tables 1 and 2 depict the means and standard deviations of the main measures employed in this study, prior and posterior beliefs, task sensitivity (d’), and metacognitive sensitivity (meta-d’) for nanotechnology and climate change, respectively. For a comparison of meta-d’ and d’ with respect to the statistical significance (3-σ-region) of respective α and γ values, see Tables 4 (nanotechnology) and 5 (climate change).

**Table 1:** Means, standard deviations, and correlations with confidence intervals for nanotechnology

**Variable**	***M***	***SD***	**Range**	**Theoretical range**	**1**	**2**	**3**
Prior belief	2.59	3.37	[-8, 8]	[-8, 8]			
Posterior belief	1.83	3.33	[-8, 8]	[-8, 8]	.51**		
					[.43, .58]		
Meta-d'	1.40	1.16	[-2.48, 4.23]	[-6.93, 6.93]	.15**	.15**	
					[.05, .25]	[.05, .25]	
d'	1.64	0.94	[-2.56, 2.56]	[-6.93, 6.93]	.06	.01	.38**
					[-.05, .16]	[-.10, .11]	[.29, .47]

*Note. M* and *SD* are used to represent mean and standard deviation, respectively. Values in square brackets indicate the 95% confidence interval for each correlation. The confidence interval is a plausible range of population correlations that could have caused the sample correlation (Cumming, 2014)

*indicates *p* < .05

**indicates *p* < .01

**Table 2:** Means, standard deviations, and correlations with confidence intervals for climate change

**Variable**	***M***	***SD***	**Range**	**Theoretical range**	**1**	**2**	**3**
Prior belief	4.36	4.33	[-8, 8]	[-8, 8]			
Posterior belief	3.26	4.59	[-8, 8]	[-8, 8]	.83**		
					[.79, .86]		
Meta-d'	1.07	0.78	[-0.80, 3.25]	[-6.93, 6.93]	.19**	.23**	
					[.09, .29]	[.13, .33]	
d'	1.52	0.78	[-1.93, 1.93]	[-6.93, 6.93]	.04	.04	.41**
					[-.06, .15]	[-.06, .14]	[.32, .49]

*Note. M* and *SD* are used to represent mean and standard deviation, respectively. Values in square brackets indicate the 95% confidence interval for each correlation. The confidence interval is a plausible range of population correlations that could have caused the sample correlation (Cumming, 2014)

*indicates *p* < .05

**indicates *p* < .01

#### Distribution of prior and posterior beliefs

**Fig. 1:**
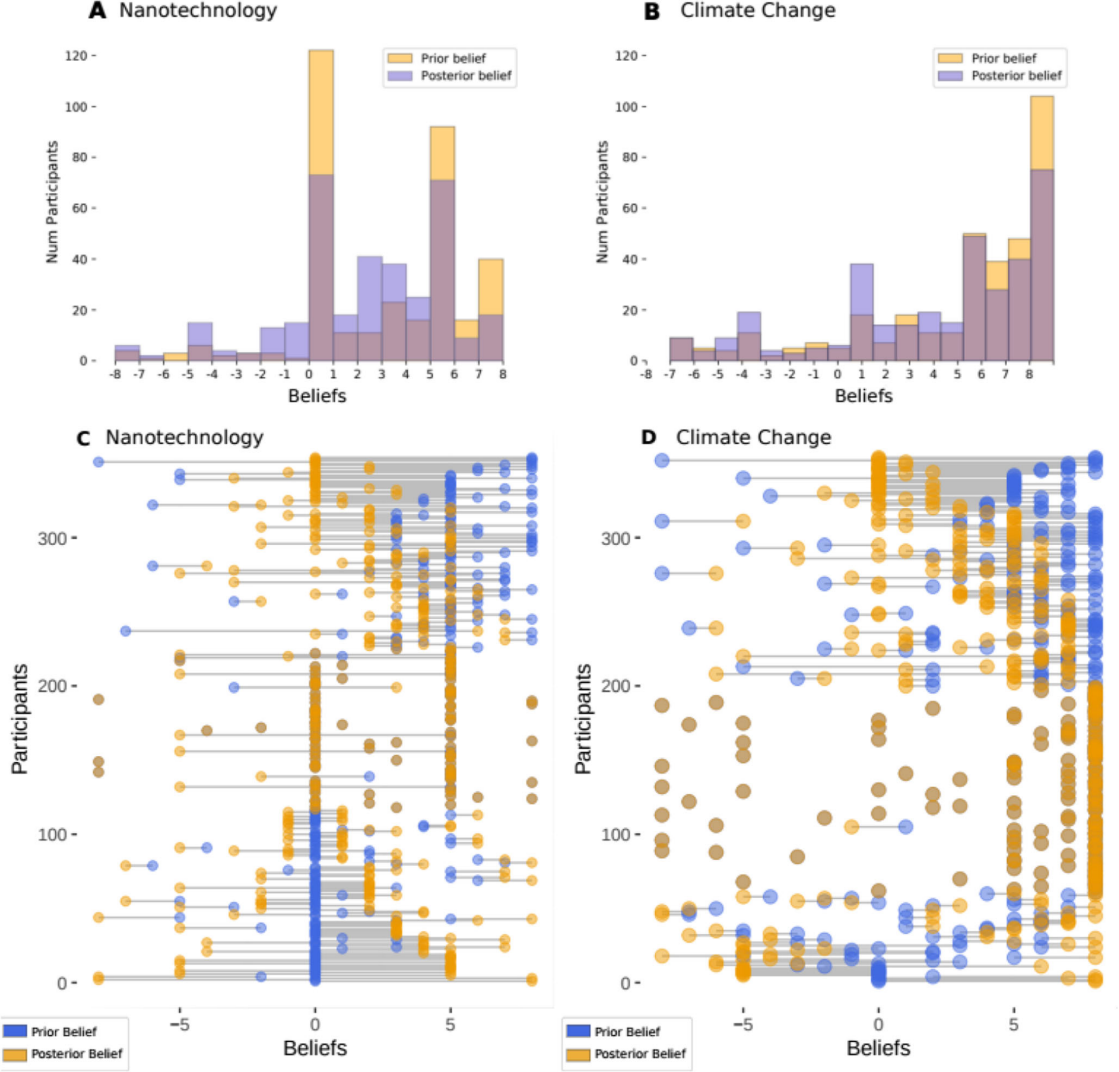
Distribution of prior and posterior beliefs. The figure displays the distribution of prior and posterior beliefs for nanotechnology (**panel A**) and climate change (**panel B**) and the direction and strength of belief updating for nanotechnology (**panel C**) and climate change (**panel D**). For climate change, more positive values indicate stronger beliefs in the anthropogenicity of climate change. For nanotechnology, more positive values indicate stronger beliefs in the benefits of nanotechnology. Panels C and D display the number of participants arranged according to the difference between their prior and posterior belief. With respect to their specific belief-updating profiles, participants could be classified into five sub-groups: (i) those whose beliefs became more moderate (displayed in the upper third of the graph); (ii) those who did not update their beliefs, and (iii) those who flipped, that is, whose posterior beliefs are of equal strength, but in the opposite direction compared to their prior beliefs (middle part of the graph); (iv) those who started from a neutral position and only developed a directed belief through the presented evidence (lower third of the graph); and (v) those whose posterior beliefs became more extreme in the direction of the prior belief, polarizers (lower third of the graph)

Figure 1 shows the overall distribution of prior and posterior beliefs (panels A and B), as well as the individual-specific shifts of prior and posterior beliefs beliefs (panels C and D). As Panels A and B show, participants held stronger prior beliefs for climate change compared to nanotechnology. Panels C and D reveal that the belief-updating distributions were fundamentally different for the two domains. Panel C (nanotechnology) displays a tree-like structure, suggesting that the majority of participants reported initially neutral views, and only formed beliefs in response to the evidence. Panel D (climate change), in contrast, displays a crescent-like structure suggesting that the majority of participants reported initially strong beliefs, and only few participants changed their views in response to the evidence.

#### Polarization fingerprints of both domains

**Fig. 2:**
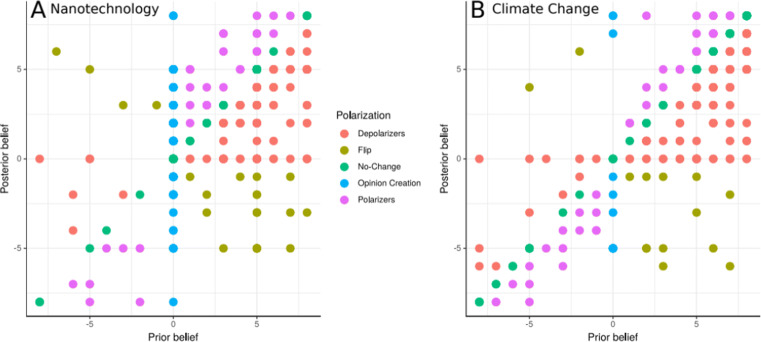
Sub-groups of participants based on their specific belief-updating profiles. The figure displays the five different sub-groups of participants, that is (i) those whose beliefs became more moderate (depolarizers, red); (ii) those who did not update their beliefs (no-change, green); (iii) those who flipped, that is, whose posterior beliefs are of equal strength, but in the opposite direction compared to their prior beliefs (flip, khaki); (iv) those who started from a neutral position and only developed a directed belief through the presented evidence (opinion creation, blue); and (v) those whose posterior beliefs became more extreme in the direction of the prior belief (polarizers, magenta). Percentages of participants for each group for nanotechnology (**panel A**) were: depolarizers = 33.33%, no-change =28.25%, flip = 6.50%, opinion creation = 23.16%, polarizers = 8.76%. Percentages of participants for each group for climate change (**panel B**) were: depolarizers = 41.52%, no-change = 38.98%, flip = 4.24%, opinion creation = 3.39%, polarizers = 11.86%

The total proportion of polarizers was higher for climate change (11.9% ± 0.63%) compared to nanotechnology (8.76% ± 0.46%). Distinct polarization “fingerprints” emerged for the two topics. For nanotechnology (Fig. 2, panel A), polarizers (pink) were almost exclusively found in one part of the spectrum of prior beliefs, namely among those who held strong prior beliefs in the benefits of nanotechnology. For climate change, in contrast, polarizers were found across the entire spectrum of prior beliefs (Fig. 2, panel B). Taken together, the present results suggest that climate change was a more polarized topic compared to nanotechnology, as found in previous research (Drummond & Fischhoff, 2017).

#### Relationship between prior beliefs and interpretation of the evidence

**Fig. 3:**
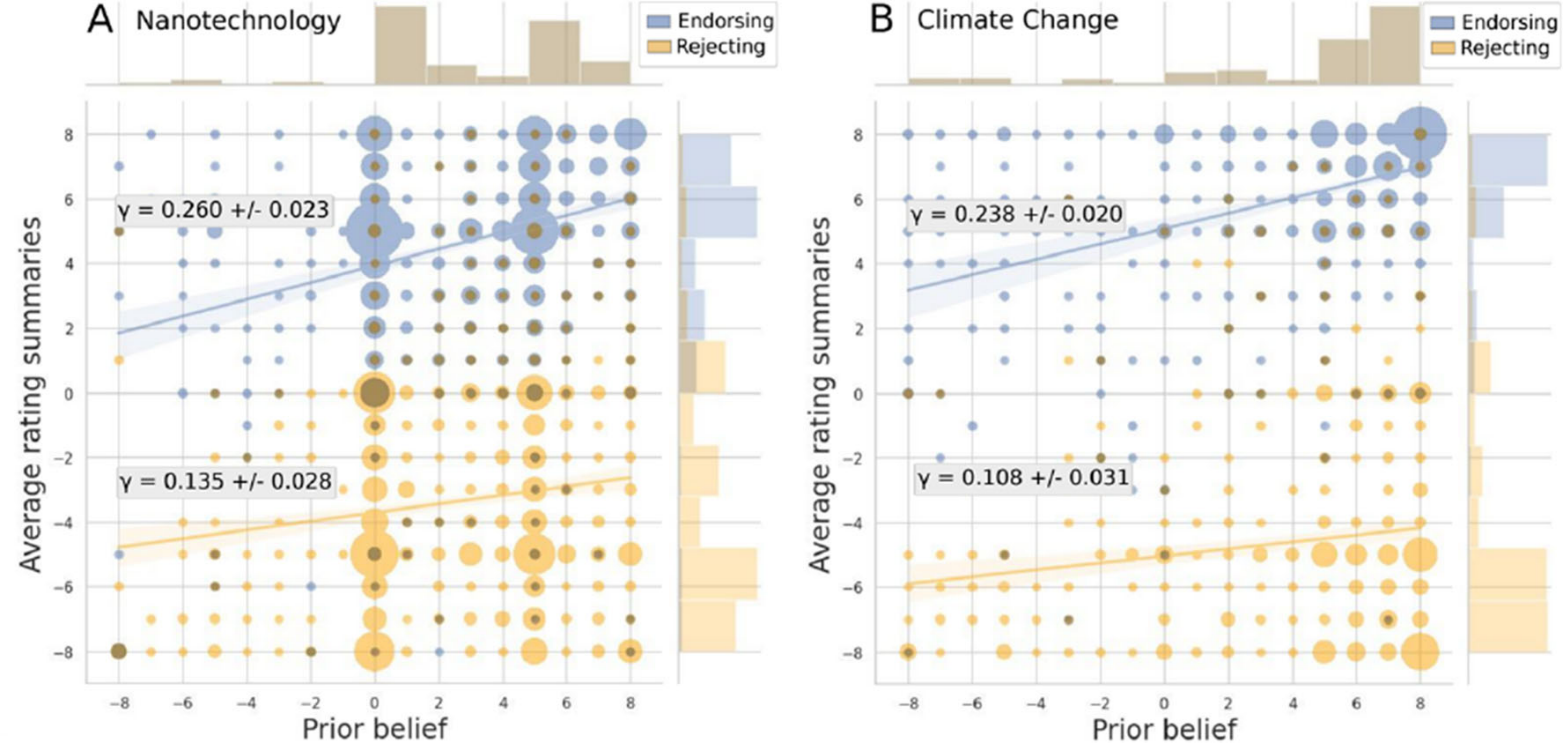
Relationship between prior beliefs and the average interpretation of the evidence. The figure displays the average interpretation for each participant for nanotechnology (**panel A**) and climate change (**panel B**), separate per direction of evidence. Endorsement of nanotechnology benefits is displayed in blue, rejection of nanotechnology benefits is displayed in yellow. Endorsement of anthropogenicity of climate change is displayed in blue, rejection of anthropogenicity of climate change is displayed in yellow. Regression lines are displayed with 95% confidence intervals. The marginal histograms show the distribution of prior beliefs (top) and of the average rating of the summaries (right). Circle sizes indicate participant numbers.

Figure 3 depicts the relationship between participants’ prior beliefs and the average rating of the direction of the evidence for nanotechnology (**panel A**) and climate change (**panel B**). The figure demonstrates, first, that participants were able to correctly classify the direction of the evidence in that endorsing evidence was rated as endorsing on average, and rejecting evidence was rated as rejecting on average. More importantly, second, the direction and strength of the influence of the prior beliefs γ on the interpretation of the evidence was positive, for both directions of evidence, and for both climate change and nanotechnology (see Table 3 for the specific values of α and γ). This result suggests that participants’ interpretations were influenced in the direction of their prior beliefs. Specifically, prior beliefs moved the interpretation of the evidence between 11% and 26% of the strength of the prior in the direction of the prior. These results align with previous research (Fryer Jr et al., 2019).

**Table 3:** Relationship between prior beliefs and interpretation of the scientific evidence for nanotechnology and climate change

Nanotechnology	α	Δ*α*	*γ*	Δ*γ*	RSE
Endorsing	3.916	0.096	0.260	0.023	2.869
Rejecting	-3.715	0.117	0.135	0.028	3.485
**Climate change**					
Endorsing	5.073	0.124	0.238	0.020	2.323
Rejecting	-5.044	0.190	0.108	0.031	3.578

*Do task sensitivity and metacognitive sensitivity relate to polarization?* We compared whether task sensitivity (d’) and metacognitive sensitivity (meta-d’) relate to polarization over the science (see Tables 4 and 5 for parameter values). Higher task sensitivity, first, was related to a lower proportion of polarizers, for both nanotechnology and climate change, suggesting that participants who were more accurate in interpreting the evidence were less likely to polarize in their beliefs. Most importantly, second, for climate change, but not for nanotechnology, the proportion of polarizers was higher among participants with lower compared to higher metacognitive sensitivity. These results suggest that higher metacognitive sensitivity was related to lower polarization for the heavily politicized topic of climate change but not to the lesser politicized topic of nanotechnology. Since metacognitive sensitivity as measured with meta-d’ controls for metacognitive bias, these results hold independently of participants’ general tendency to report high/low values of confidence.

**Table 4:** Relationship between metacognitive sensitivity (meta-d’) and task sensitivity (d’) and parameters (i) α (mean interpretation of evidence), (ii) γ (relationship between prior beliefs and interpretation of evidence), and (iii) the proportion of polarizers for nanotechnology

**NT**	**meta-d’**					
	**Low scorer (n = 198)**			**High scorer (n =156)**		
	α±Δα	γ±Δγ	RSE	α±Δα	γ±Δγ	RSE
End.	2.925±0.132*	0.353±0.034*	3.057	5.430±0.114*	0.091±0.025*	2.144
Rej.	-3.162±0.149*	0.232±0.038	3.443	-4.574±0.174*	0.096±0.038	3.270
	Pol±ΔPol(%): 9.60±0.68			Pol±ΔPol(%): 7.70±0.62		
	**d’**					
	**Low scorer (n = 126)**			**High scorer (n = 228)**		
	α±Δα	γ±Δγ	RSE	α±Δα	γ±Δγ	RSE
End.	2.185±0.192*	0.514±0.045*	3.431	4.892±0.090*	0.112±0.021*	2.155
Rej.	-2.113±0.210*	0.304±0.049*	3.752	-4.587±0.113*	0.036±0.027*	2.688
	Pol±ΔPol(%): 14.3±1.27*			Pol±ΔPol(%): 5.70±0.38*		

*Note*. Significant differences between high and low scorers (3-σ-region) are denoted with *

**Table 5:** Relationship between metacognitive sensitivity (meta-d’) and task sensitivity (d’) and parameters (i) α (mean interpretation of evidence), (ii) γ (relationship between prior beliefs and interpretation of evidence), and (iii) the proportion of polarizers for climate change

**CC**	**meta-d’**					
	**Low scorer (n = 195)**			**High scorer (n = 159 )**		
	α±Δα	γ±Δγ	RSE	α±Δα	γ±Δγ	RSE
End.	4.119±0.172*	0.296±0.03*	2.602	6.815±0.120*	0.073±0.018*	1.318
Rej.	-4.408±0.255*	0.210±0.044	3.863	-6.032±0.242*	0.057±0.037	2.669
	Pol±ΔPol(%): 17.9±1.30*			Pol±ΔPol(%): 4.40±0.35*		
	**d’**					
	**Low scorer (n =93)**			**High scorer (n = 261)**		
	α±Δα	γ±Δγ	RSE	α±Δα	γ±Δγ	RSE
End.	2.218±0.298*	0.535±0.049*	2.889	6.114±0.096*	0.129±0.016*	1.533
Rej.	-2.156±0.440*	0.330±0.072*	4.265	-6.096±0.114*	0.036±0.019*	1.834
	Pol±ΔPol(%): 19.4±2.01*			Pol±ΔPol(%): 9.19±0.57*		

*Note*. Significant differences between high- and low-scorer (3-σ-region) are denoted with *.

For both topics, participants with lower metacognitive sensitivity rated the summaries as less strong evidence than participants with higher metacognitive sensitivity. At the same time, the influence of their prior beliefs was stronger for lower metacognitive sensitivity. Specifically, participants with low metacognitive sensitivity rated evidence disproving their prior beliefs as almost neutral or (given particularly strong priors) even as confirming their prior.

*Does metacognitive sensitivity (meta-d’) moderate the relationship between prior beliefs and evidence interpretation?* To explore the anticipated cognitive process behind the effect that metacognitive sensitivity relates to polarization, we investigated whether metacognitive sensitivity (meta-d’) moderates the relationship between participants’ prior beliefs and their interpretation of the scientific evidence.

**Fig. 4:**
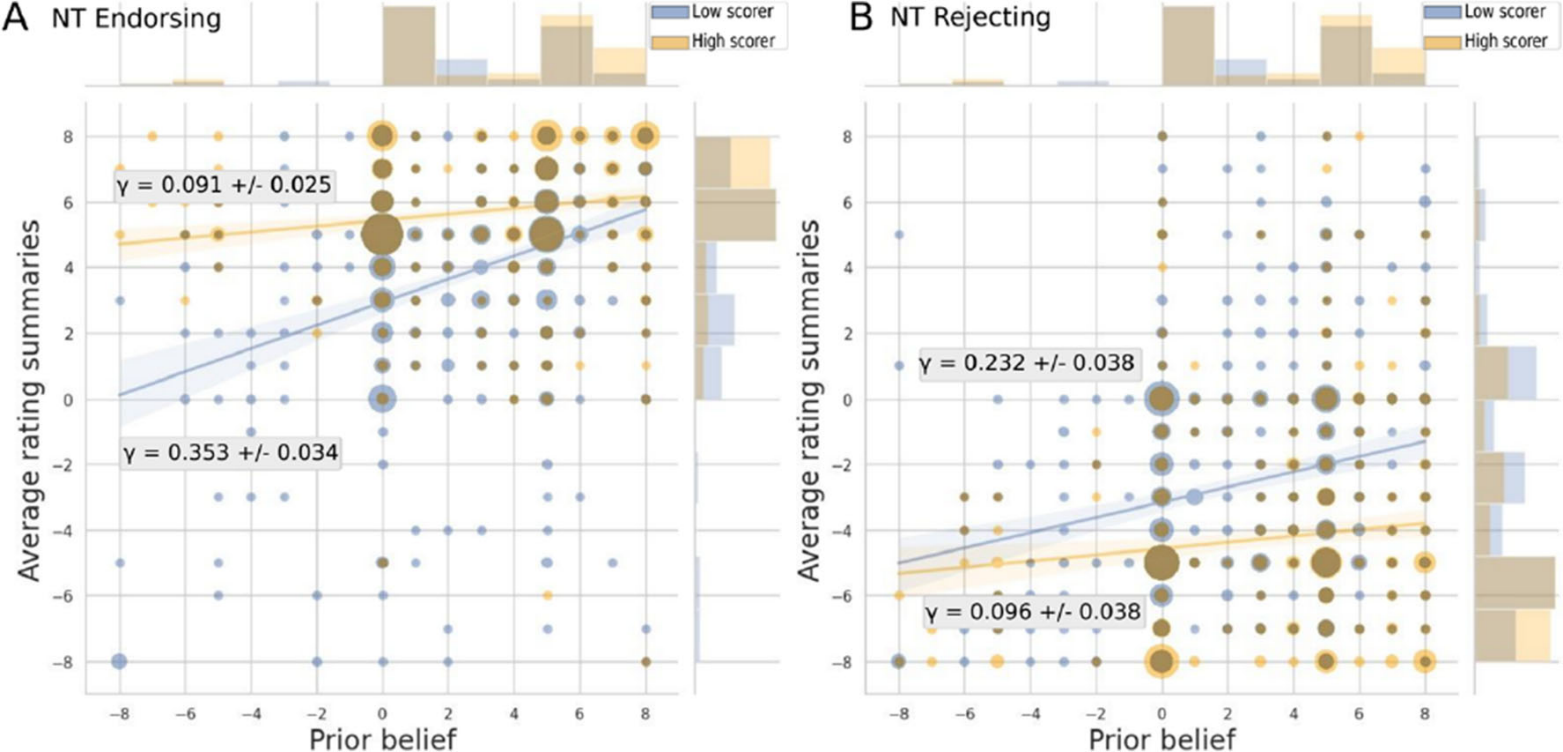
Moderating influence of metacognitive sensitivity (meta-d’) on the relationship between prior beliefs and interpretations of the scientific evidence for nanotechnology. The figure displays the relationship between prior beliefs and interpretations of the scientific evidence, separately for participants high (yellow) compared to participants low (blue) in metacognitive sensitivity (meta-d’), and separately per direction of the evidence (endorsing, **Panel A** vs. rejecting, **Panel B**). Regression lines are displayed with 95% confidence intervals. The marginal histograms show the distribution of prior beliefs (top) and the average interpretation of the summaries (right). Circle sizes indicate participant numbers.

Figure 4 suggests that for nanotechnology higher meta-d’ attenuated the relationship between prior beliefs and the interpretation of the evidence for evidence endorsing nanotechnology benefits: For participants with low metacognitive sensitivity, the relationship between prior beliefs and evidence interpretation was higher for the positive direction of evidence (endorsing) γ_NT_Endorsement_ = 0.353±0.034 compared to participants with high metacognitive sensitivity γ_NT_Endorsement_ = 0.091±0.025. For participants with low metacognitive sensitivity the relationship between prior beliefs and evidence interpretation was not significantly different for the negative direction of evidence (rejecting) γ_NT_ Rejection_ = 0.232±0.038 compared to the relationship for participants with high metacognitive sensitivity γ_NT_Rejection_= 0.096±0.038.

Furthermore, we calculated the correlation between metacognitive sensitivity and summary ratings for participants high and low in metacognitive sensitivity. For participants with low metacognitive sensitivity, there was a significant relationship for the positive direction of evidence (endorsing): r_NT_Endorsement_(790) = .24, 95%CI = [.17, .30], and for the negative direction of evidence (rejecting): r_NT_Rejection_(790) = -.16, 95%CI = [-.23, -.09]. For participants high in metacognitive sensitivity, the relationship between prior beliefs and the interpretation of the evidence was virtually non-existent for the positive direction of evidence (endorsing): r_NT_Endorsement_(622) = .01, 95%CI = [-.02, .17]. However, for the negative direction of evidence (rejecting) the strength of the relationship was similar to that of participants with low metacognitive sensitivity: r_NT_Rejection_(622) = -.15, 95%CI = [-.23, -.08].

**Fig. 5:**
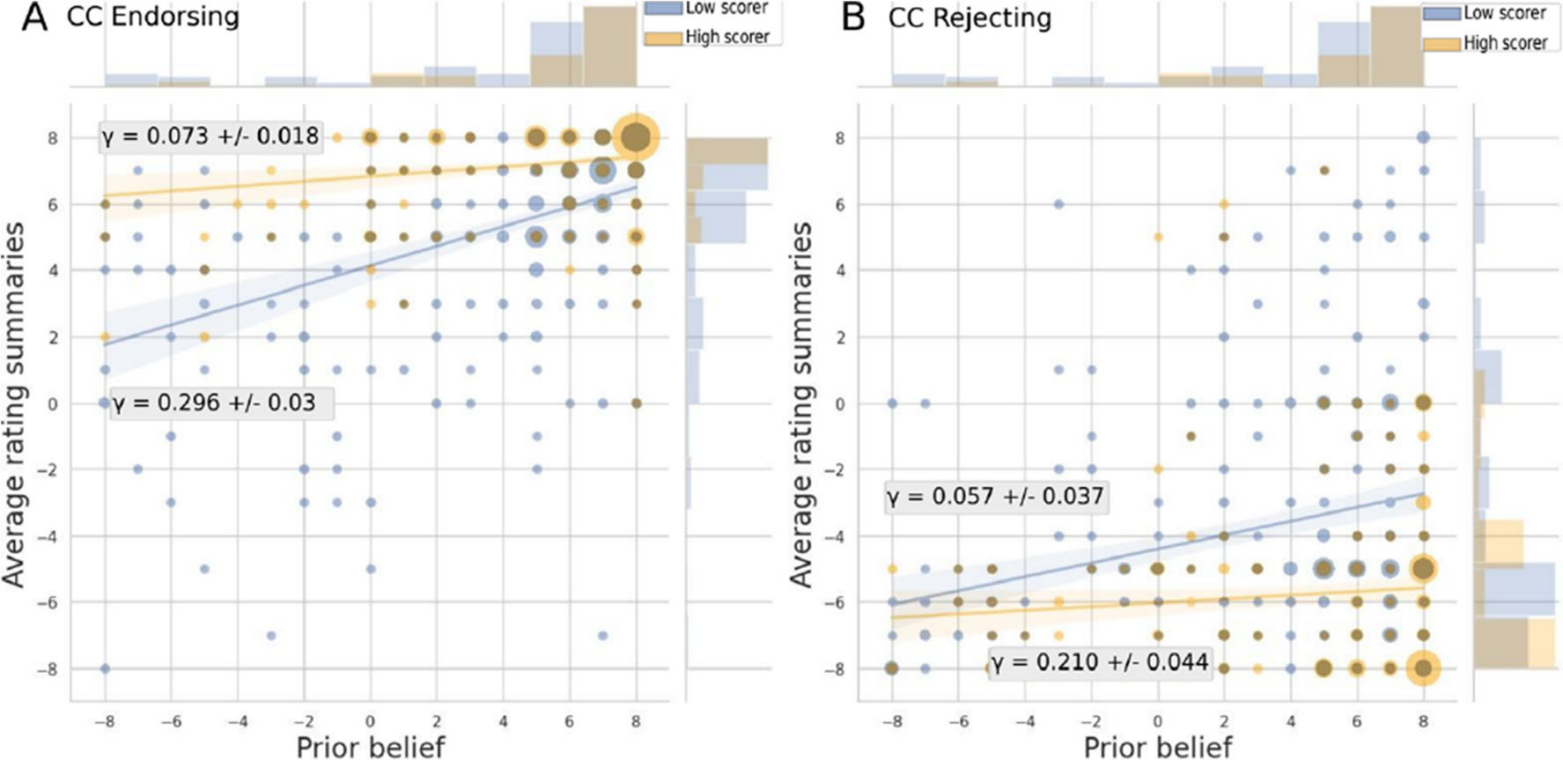
Moderating influence of metacognitive sensitivity (meta-d’) on the relationship between prior beliefs and interpretations of the scientific evidence for climate change. The figure displays the relationship between prior beliefs and interpretations of the scientific evidence, separately for participants high (yellow) compared to participants low (blue) in metacognitive sensitivity (meta-d’), and separately per direction of the evidence (endorsing, **Panel A** vs. rejecting, **Panel B**). Regression lines are displayed with 95% confidence intervals. The marginal histograms show the distribution of prior beliefs (top) and the average interpretation of the summaries (right). Circle sizes indicate participant numbers.

Similarly, Fig. 5 suggests that for climate change higher meta-d’ attenuated the relationship between prior beliefs and the interpretation of the evidence for evidence endorsing anthropogenicity of climate change: For participants with low metacognitive sensitivity the relationship between prior beliefs and evidence interpretation was higher for the positive direction of evidence (endorsing) γ_CC_Endorsement_ = 0.296±0.03 compared to participants with high metacognitive sensitivity γ_CC_Endorsement_ = 0.073±0.018. For participants with low metacognitive sensitivity the relationship between prior beliefs and evidence interpretation was not significantly different for the negative direction of evidence (rejecting) γ_CC_ Rejection_ = 0.210±0.044 compared to the relationship for participants with high metacognitive sensitivity γ_CC_Rejection_= 0.057±0.037. Furthermore, we calculated the correlation between metacognitive sensitivity and summary ratings for participants high and low in metacognitive sensitivity. Results corroborate our findings in that: for participants with low metacognitive sensitivity, there was a significant relationship for the positive direction of evidence (endorsing): r_CC_Endorsement_(388) = .36, 95%CI = [.27, .44] and for the negative direction of evidence (rejecting): r_CC_Rejection_(388) = -.21, 95%CI = [-.31, -.12]). For participants high in metacognitive sensitivity, there was a low relationship for the positive direction of evidence (endorsing): r_CC_Endorsement_(316) = .16, 95%CI = [.05, .27] and for the negative direction of evidence (rejection) it was virtually non-existent: r_CC_Rejection_(316) = -.09, 95%CI=[-.20, 0.02].

However, for the negative direction of evidence (rejecting) the difference between correlation coefficients for participants low and participants high in metacognitive sensitivity was non-significant.

That is, for nanotechnology and climate change, metacognitive sensitivity moderated the influence of the prior for the positive direction of evidence (endorsing) but not for the negative direction of evidence (rejecting). Metacognitive sensitivity moderating the influence of the prior for the positive (but not negative) direction of evidence only might be due to very low numbers of participants holding strong initial beliefs in line with the negative direction of evidence (2.2% for nanotechnology and 5.1% for climate change).

## Discussion

Civil polarization has long been the domain of political and religious worldviews. Recent years, however, have seen a marked increase in the polarization over seemingly emotionless science such as climate change, vaccination, COVID-19, or nanotechnology. Leveraging methods from Signal Detection Theory, we found that individuals with higher metacognitive sensitivity (measured as *meta-d’*), an insight into the validity and fallibility of own interpretations of the available evidence, were less likely to polarize over the heavily contested topic of climate change, but not the less heavily contested topic of nanotechnology. Specifically, among citizens with lower metacognitive sensitivity, the proportion of polarizers over climate change was four times higher than among citizens with higher metacognitive sensitivity.

Furthermore, the present research also isolated a cognitive mechanism that explains *why* metacognitive insight can relate to polarization over contested science. Prior research has established a link between the degree of politicization of different scientific domains, and the accuracy of metacognitive insight in that domain (Fischer et al., 2019). Furthermore, emerging evidence suggests a relationship between metacognitive insight, and the accuracy of evidence accumulation, and belief updating (Fischer & Said, 2021; Sinclair et al., 2020). Based on this evidence, we expected that metacognitive insight into the accuracy of one’s interpretation of scientific evidence might relate to belief-updating about politicized science. Indeed, results suggest that lower insight into the accuracy of own interpretations of the available evidence fostered updating in the direction of strong prior beliefs (at the expense of the evidence); conversely, higher metacognitive insight fostered belief-updating in the direction of the evidence (at the expense of prior beliefs).

The present research found an influence of metacognition for the heavily politicized topic of climate change, but not for the less heavily politicized topic of nanotechnology. The two topics differed in that (i) the prior belief distributions of both topics were markedly different in that more participants started out with neutral views on nanotechnology compared to climate change; and (ii) the polarization fingerprints of both topics differed in that, for nanotechnology, participants who polarized were found almost exclusively among endorsers of nanotechnology benefits, whereas for climate change, participants polarized across the whole belief spectrum. Furthermore, metacognitive sensitivity moderated the relationship between prior beliefs and interpretation for endorsers of nanotechnology benefits/anthropogenicity of climate change, that is, only where participants held initially strong beliefs.

These results highlight a novel factor that relates to a lower likelihood of polarization over contested science: metacognitive insight into the accuracy of interpretations of the available evidence. This may be particularly relevant in areas of politicized science where external information on the accuracy of information is noisy in that false and misleading information exists alongside information that is scientifically accurate. In noisy decision environments lacking unequivocal external feedback about the reliability of information, citizens might be particularly prone to relying on internal cues to accuracy such as metacognitive confidence (Desender et al., 2018; Rahnev et al., 2015). In line with a mechanism whereby the degree of politicization, and hence noisiness, of the decision environment affects the degree of reliance on internal, metacognitive cues for belief-updating, the present results found a moderating influence of metacognitive insight for climate change, but not nanotechnology.

The present study has limitations. First, given the low trial numbers, metacognitive sensitivity estimates may have low precision, and may be dependent on the particular characteristics of the stimuli used. However, we used a hierarchical Bayesian approach for estimation of metacognitive sensitivity, as this approach produces reliable results in case of low trial numbers (at least in the case of group estimates; Fleming, 2017). Furthermore, low precision of estimates appears to be particularly pronounced for cases of high task sensitivity (d’=2), where metacognitive sensitivity tends to be overestimated (Fleming, 2017). In the present case, mean task sensitivity was 1.64 for nanotechnology and 1.52 for climate change. For nanotechnology, about one-third of participants had a task sensitivity of 2 or higher, while for climate change, task sensitivity was below 2 for *all* participants. Therefore, metacognitive sensitivity estimated for nanotechnology might constitute an overestimation, but likely constitutes a reliable estimate for climate change.

Second, the comparability between both topics, climate change and nanotechnology, may be limited since the number of trials and wording of belief items differed between the two topics. Specifically, for nanotechnology, participants indicated whether they believe that the benefits of nanotechnology outweigh the risks, whereas for climate change, participants indicated whether they believe that climate change is anthropogenic. Hence, the assessed beliefs are markedly different, and being asked about anthropogenicity versus risks likely triggers different cognitive associations. However, both beliefs were chosen to be in line with prior literature on these topics (Drummond & Fischhoff, 2017; Fryer Jr et al., 2019), and to reflect central aspects in the public debate about each topic (anthropogenicity for climate change, and risks for nanotechnology; Hamilton et al., 2015; Macoubrie, 2004; Wright, 2016). Most importantly, however, the present results aimed at understanding the relationship between metacognitive sensitivity, interpretation of evidence, and belief-polarization irrespective of content, and *within* each topic. Low comparability *between* topics should therefore not affect the validity of these results. However, future research could benefit from a more formal assessment of the generalizability of our results by assessing identical beliefs (e.g., climate change, vaccination, and nanotechnology risks), and by comparing a wider range of contested and less contested topics across the whole spectrum of scientific domains.

Third, participants indicated their interpretation of the evidence on a 17-point scale as opposed to making a binary judgment as in typical SDT experiments. While interpretations of evidence were always binary-coded for SDT analyses, and hence did not reflect the strength of the answer, participants may still have perceived the answer as less clear-cut than they would have when prompted to give a binary judgment. In particular, participants may perceive the judgment of whether a study endorses, say, nanotechnology risks, as more of a graded one (as opposed to having a clear TRUE/FALSE answer). This may have reduced the extent of subsequent belief-updating: When participants perceive the encountered evidence as more open to interpretation (vs. unequivocal), they may be less likely to update their beliefs. In line with this reasoning, it was found that communicating scientific consensus on climate change affects the beliefs citizens hold about the issue (for a review, see Van der Linden, 2021). Hence, the extent of belief-updating observed in the present study may constitute an underestimation.

Human interpretations of evidence about the external world are imperfect: We tend to mis-interpret evidence asymmetrically as a function of prior beliefs (Corner et al., 2012; Fryer Jr et al., 2019), worldviews (Kahan et al., 2017), or partisanship (for a meta-analysis, see Ditto et al., 2019). Metacognitive sensitivity, however, enables us to do justice to this fundamental inclination by evaluating varying levels of accuracy of interpretation with matching levels of confidence. This process of stepping back from own interpretations has been described as an “internal tribunal” that rules on the soundness of own interpretations (Fleming, 2014). Here, participants with more accurate “internal tribunals” were less likely to view evidence through the lens of their own prior beliefs.

Recent research has connected cognitive reflection to correct assessment of information, such as the ability of citizens to discriminate between real and fake news (Pennycook & Rand, 2019) as well as misperceptions about politicized science (e.g., COVID-19: Pennycook, McPhetres, Bago, & Rand, 2021). While these studies focused on how cognitive reflection is related to reasoning of the subject matter itself, the present study demonstrates the importance of metacognitive reflection, citizens’ insight into the accuracy of their reasoning. However, as both measures are concerned with citizens’ reflective ability, one might expect both object-level reasoning (assessed with the CRT or numeracy) and metacognitive sensitivity (meta-d’) to be somewhat related. Future research could investigate how object-level and metacognitive reflective abilities jointly shape citizens’ reasoning about, or behavior in relation to, contested science (Fischer, Huff & Said, 2021a).

The present metacognitive review offers an interesting leverage point to address societal polarization over contested science. Specifically, interventions targeted at increasing metacognitive accuracy (Elosúa et al., 2013; Zohar & Barzilai, 2013) may provide an effective means to mitigate societal polarization over science. This is encouraging since reducing political polarization is particularly relevant for domains where unified, collective action is required, such as for combating climate change (Brechin, 2016; Harris, 2007), but where prior research has repeatedly demonstrated that simply increasing the accuracy of object-level cognition, such as scientific literacy, or numeracy has limited success in reducing societal polarization, and may in fact even propel a societal divide (Kahan et al., 2012, 2017; but see Fischer, Huff & Said, 2021b).

To conclude, metacognitive insight into interpretations of scientific evidence is related to a lower likelihood to polarize over contested science. Undifferentiated faith in own interpretations of scientific evidence drives polarization over contested science, while more nuanced insight into the limitations and fallibility of own interpretations reduces polarization over contested science. By demonstrating the impact of metacognition, these results increase our understanding of the mechanisms that may link metacognition and polarization over contested science.

## Supplementary Information

ESM 1 (PDF 38 kb)

## Footnotes

1 For climate change, participants also read two neutral texts. Since these could not be rated as endorsement or rejection of anthropogenicity, however, they were excluded from the analysis.
