# Supplementary material for: Contested science: Individuals with higher metacognitive insight into interpretation of evidence are less likely to polarize
Source: Psychon Bull Rev. 2021 Oct 29;29(2):668–80. doi: 10.3758/s13423-021-01993-y (PMC8555729; doi:10.3758/s13423-021-01993-y)
Supplement: Supplementary file 1 — (PDF 38 kb) [file 13423_2021_1993_MOESM1_ESM.pdf]

Supplementary Material

Nanotechnology

Endorsement

Gref, Couvreur & Mysiakine (2003)

In this study the engineering of a ligand-coupled nanoparticle is described. Such particles can be used to direct medication to specific parts of the human body and thus perform non-invasive treatment methods.

The researchers found that the particle they synthesized was not toxic towards cells. Even with very high concentrations of the particle there was no visible effect for over 80% of the investigated cells. Additionally, the authors were able to prove that the particle was able to couple onto different drug components.

This suggests that ligand-coupled nanoparticle could be used for low risk future medication methods.

Liu, Tang & Ai (2003)

In this study the authors investigate the effect of hydroxiapatie (HAP) nanoparticles on human liver cancer cells. The benefits of such particles lie in fighting cancer cells and thus in a clinical application.

The researchers found that the treatment of liver cancer cells with the nanoparticles induced a dose-dependent growth inhibition and induction of programmed cell death.

This suggests that HAP nanoparticles could be used for the treatment of liver cancer in the future.

Rajagopalan et al. (1996)

In this study the authors analyse the pharmacological effect of fullerene nanoparticles. Such particles can be used in a wide variety of fields. For example, such particles are tested in the treatment of HIV.

The researchers found that there is a therapeutic window in which the particle lowered the activity of the HI-virus with only minimal side effects on human cells.

This suggests that fullerenes might play a major role in the treatment of aids patients and fighting HIV.

Schulz et al. (2002)

In this study the authors analyse the effects of titanium dioxide and zinc oxide nanoparticles on the human skin. The use of such particles is for example in the application of modern sun cream with a vastly enhanced UV-protection.

The researchers found that neither surface characteristics, particle size nor shape of the micronized particles result in any dermal absorption of the particles. Instead the particles are deposited on the surface of the skin.

This suggests that the application of such particles in cream does not hold risks for the skin.

<u>Rejection</u>

ETC Group (2003)

This study is a meta-analysis about the influence of nanotechnology on science, technology, politics, society and ethics.

Researchers investigated different sources reaching back to the eighties. In conclusion they came to a rather negative stance on nanotechnology. The authors justified this by pointing out the ecological consequences of the technology. In result they asked for a freeze of research for commercial products based on nanotechnology.

In conclusion, the authors were skeptical towards the consequences of nanotechnology on environment and society.

Lam et al. (2003)

In this study the authors investigated the connection between single-walled-carbon-nanotubes (SWCNT) and inflammation of the lungs. The research focused on the particle in airborne form. The benefits of such a particle lie in its extreme tensile strength and stability which leads to interesting applications as an industrial material.

The researchers analyzed three variants of the particle and found that it not only accumulated in dead but also living lung tissue. Furthermore, it induced programmed cell death in a significant amount of those cells.

This suggests that an exposition with SWCNT is a risk that should not be neglected.

Oberdörster (2000)

In this study the authors looked into the existing studies on ultrafine nanoparticles. Such particles can be used in the production of new materials for industry. Such materials often have – because of their special surface properties – an enhanced durability.

The researchers found that ultrafine nanoparticles administered to the lung cause a greater inflammatory response than do larger particles. Additionally it was shown that such particles can cross the blood-brain barrier and can be transported to the brain from the lung.

This suggests that the use of ultrafine nanoparticles for industrial purpose is associated with risk.

Wahrheit et al. (2004)

In this study the toxicological effect of single-walled carbon nanotubes (SWCNT) is analysed. The focus of the research is on the particles effect on the lung. The benefits of such particles for the industry are their extreme stability and tensile strength.

The researchers found that such particles could block the respiratory tract. This was the main toxicological effect that was found in the study. Additionally inflammation effects of such particles are found.

This suggests that if SWCNT particles are used in the industry they have to be handled with caution and exposition at the workspace has to be limited.

Climate Change

Endorsement

Hansen et al. (1981)

The average global temperature rose by 0.2°C between the mid1960s and 1980. When humans burn fuels like coal and oil to make power, it releases carbon dioxide into the atmosphere.

Scientists have created a model to predict how much the temperature should rise for each ton of carbon dioxide released into the atmosphere. The increases in temperature between 1960 and 1980 closely matched the predictions. As the amount of carbon dioxide released increased, so did the temperature. Although the actual temperatures were a little different from the predictions, these differences can be explained by volcanic eruptions and other factors.

Thus, the scientists conclude that the carbon dioxide released into the atmosphere by man is increasing world temperatures.

National Climatic Data Center (2009)

This paper describes how changes in temperatures are affecting the world. Average global temperatures on the surface of both water and land have gone up by close to 1.4°F since the early 1900s. Average temperatures on the surface of both water and land in the U.S. have also gone up by about 2°F during that same period.

Furthermore, we know that these changes are the result of carbon dioxide released by humans. Scientists created computer models to predict how much temperatures would rise as more carbon dioxide was released into the atmosphere. The actual changes in temperatures have closely matched the predictions, suggesting that carbon dioxide is causing temperatures to rise.

In conclusion, the record amount of carbon dioxide in the atmosphere and the dramatic increase in temperatures around the world suggest that these changes are the result of human activity.

Rejection

Moberg et al. (2005)

In this paper, scientists used data from tree rings and other sources to estimate temperatures over time. You can estimate temperatures from tree rings since higher temperatures increase tree growth and lead to larger tree rings.

The scientists worked out that there were two very warm periods in the past: 1000-1100 AD and around 1600 AD. In these years, the temperatures were about the same as they were between 1960 and 1990. Humans were not burning large amounts of fossil fuels in 1000 AD or 1600 AD. Thus it seems that those older warm periods were caused by natural ups-and-downs in the world's climate, not by humans.

Thus the evidence suggests that the current warm period we are experiencing is also caused by natural ups-and-downs in the world's climate and not by human activity.

Soon & Baliunas (2003)

In this paper, the scientists look at the climate over the past 1,000 years. They look at historical records, ice cores, glaciers, tree rings, lake fossils, and seafloor sediments to work out what the climate was like in each year.

The authors look for "weather anomalies." They defined a weather anomaly as a period of over 50 years where it was unusually hot or cold, wet or dry. The authors find many of these anomalies in the past. It seems unlikely that these anomalies were caused by humans, as humans did not burn large amounts of fuels, such as oil and gas, back then. These scientists believe that we are also experiencing a weather anomaly currently—it is unusually hot. However, it is not the most extreme anomaly that the authors have found; other periods have had more extreme high or low temperatures.

Thus, the current high temperatures may be caused by the global climate's natural ups-and-downs, not by human activity.
